# Analysis of Ionizing Radiation Induced DNA Damage by Superresolution dSTORM Microscopy

**DOI:** 10.3389/pore.2021.1609971

**Published:** 2021-11-08

**Authors:** Szilvia Brunner, Dániel Varga, Renáta Bozó, Róbert Polanek, Tünde Tőkés, Emília Rita Szabó, Réka Molnár, Nikolett Gémes, Gábor J. Szebeni, László G. Puskás, Miklós Erdélyi, Katalin Hideghéty

**Affiliations:** ^1^ Biomedical Applications Group, ELI-ALPS Research Institute, ELI-HU Non-Profit Ltd., Szeged, Hungary; ^2^ Department of Optics and Quantum Electronics, University of Szeged, Szeged, Hungary; ^3^ Department of Dermatology and Allergology, University of Szeged, Szeged, Hungary; ^4^ Department of Oncotherapy, University of Szeged, Szeged, Hungary; ^5^ Laboratory of Functional Genomics, Biological Research Centre, Szeged, Hungary

**Keywords:** ionizing radiation, γ-H2AX, DNA-DSB, confocal microscopy, dSTORM, superresolution

## Abstract

The quantitative detection of radiation caused DNA double-strand breaks (DSB) by immunostained γ-H2AX foci using direct stochastic optical reconstruction microscopy (dSTORM) provides a deeper insight into the DNA repair process at nanoscale in a time-dependent manner. Glioblastoma (U251) cells were irradiated with 250 keV X-ray at 0, 2, 5, 8 Gy dose levels. Cell cycle phase distribution and apoptosis of U251 cells upon irradiation was assayed by flow cytometry. We studied the density, topology and volume of the γ-H2AX foci with 3D confocal microscopy and the dSTORM superresolution method. A pronounced increase in γ-H2AX foci and cluster density was detected by 3D confocal microscopy after 2 Gy, at 30 min postirradiation, but both returned to the control level at 24 h. Meanwhile, at 24 h a considerable amount of residual foci could be measured from 5 Gy, which returned to the normal level 48 h later. The dSTORM based γ-H2AX analysis revealed that the micron-sized γ-H2AX foci are composed of distinct smaller units with a few tens of nanometers. The density of these clusters, the epitope number and the dynamics of γ-H2AX foci loss could be analyzed. Our findings suggest a discrete level of repair enzyme capacity and the restart of the repair process for the residual DSBs, even beyond 24 h. The dSTORM superresolution technique provides a higher precision over 3D confocal microscopy to study radiation induced γ-H2AX foci and molecular rearrangements during the repair process, opening a novel perspective for radiation research.

## Introduction

Radiotherapy is an important pillar of cancer management. The main aim of using ionizing radiation is to diminish cancer cells with high efficacy and selectivity. The latest technical developments provide controlled, highly conformal radiation delivery to the target, which enables the implementation of altered hypofractionation schemes with a fraction size of 3–20 Gy, changing the biological effects remarkably over the 1.8 to 2 Gy, conventionally fractionated radiotherapy. Therefore, fundamental radiobiology research is essential for the investigation of molecular processes in a time dependent manner on doses >2 Gy/fraction.

In radiobiology, the classical cell survival assays, such as MTT, MTS, SBRT, and clonogenic assays are the gold standard end points for studying cellular response to ionizing radiation (IR) with different parameters [1, 2]. Clonogenic assay is used to quantify the ability of cells to proliferate in a dose dependent manner following IR. If the cells have proliferation capacity, they grow into cellular aggregates, retaining their reproductive ability to form large colonies [3]. In recent years, advanced methods of molecular research have enabled the study of IR related subcellular changes. Molecular radiobiology has revealed radiation induced clustered DNA damage, i.e. the complex arrangement of two or more lesions (single- and double-strand breaks) within one to two helical turns of DNA. This clustered DNA damage compromises the base excision repair pathway, resulting in an increased lifetime of the lesions [4, 5]. The most severe form of such damage is double strand breaks (DSB), which threaten the genetic and epigenetic integrity of cells and can be highly lethal [6]. The activation of the complex DNA damage response (DDR) machinery starts by the signaling of DSBs, arresting the cell cycle, and triggering the DNA repair pathways [7–11]. Religation of the broken DNA ends, i.e., non-homologous end joining (NHEJ), is the predominant repair process which occurs with fast kinetics. However, more complex DSBs cannot be perfectly repaired by NHEJ. In these cases, homologous recombination (HR), a slow, but precise process using the undamaged DNA sequence as a template to reproduce the lost or changed molecules, may repair more serious DSBs [12, 13].

The degree of DSBs occurring and the kinetics of the repair process depend on the different radiation doses, the Linear Energy Transfer of radiation, the cell type and the radio-sensitivity of the cell [14]. For the quantitative assessment of DSB serine-139, phosphorylated histones H2AX (γ-H2AX) immunofluorescence staining and microscopic assessment is an established technique. The H2AX variant of histone H2A is present in subsets of nucleosomes (2–25% of the total H2A) and is implicated in DSB repair [15, 16]. When H2AX is phosphorylated at the serine residue 139 by phosphoinositide-3-kinase-related protein kinases (PIKKs), the phosphate group adopts a γ position in the protein, constituting the γ-H2AX configuration. This phosphoprotein acts in the early events of DNA repair by decondensing the chromatin near the DSB [17]. Additionally, γ-H2AX histones attach to the DSB ends, forming a “γ-H2AX focus,” which extends to several Mb at the sides of the DSB. This method enables the localization of double-strand breaks in the nucleus, and the analysis of the repair kinetics [15, 18].

The number of γ-H2AX foci increases 1 h after exposure to X-rays and then decreases slowly over time. The rate of loss of foci and the presence of residual foci correlate with cellular radiosensitivity [19–21].

Unrepaired DNA damage in the surviving cells leads to serious cellular functional deterioration and genomic disintegration, as well as to chromatid and chromosomal aberrations. Residual γ-H2AX foci may represent a promising biomarker to predict tumor radiosensitivity [22, 23].

To date, few methods and techniques have been applied to follow the changes in γ-H2AX signal intensity. Among optical methods, immunofluorescence microscopy is the most widely used technique. Confocal microscopy with Z scanning and 3D reconstruction of the entire nucleus has recently been applied for the analysis of the number of foci and the detection of the spatial distribution of DSBs. Despite the fact that its diffraction limited spatial resolution (≈λ/2) precludes the study of the substructures of the repair foci, this method can still be applied for semi-quantitative evaluation. Such studies are typically based on cluster analysis, which is able to determine the number and the volume of clusters inside the nuclei. However, due to the inherent limited spatial resolution, quantification of the repair foci is difficult, and finding the appropriate merit function is a challenge. A control method capable of determining the size and distribution of repair foci with a higher precision would be useful to calibrate the confocal results.

It was previously demonstrated that the dSTORM superresolution technique can be used to spatially resolve the substructure of repair foci and statistically determine the number of labeled γ-H2AX [24]. The dSTORM microscopy method detects individual blinking events which can be associated with spatially and temporally isolated fluorescent molecules. The response function of the imaging system (the average number of localizations belonging to a single labeled histone molecule) depends on the lifetime of the fluorescence ON state, the labeling density and the number of reactivation cycles of the applied dye molecules. These parameters can be determined by a rigorous calibration process as was discussed earlier [25–27].

In this study we aimed to get a deeper insight–by using dSTORM microscopy–into γ-H2AX distribution and dynamics at different time points after X-ray irradiation up to 5 Gy single dose of U251 (Glioblastoma Multiforme GBM) cells, and to evaluate the superresolution technique in radiobiology research.

## Materials and Methods

### Cell Line and Culture Condition

Human glioblastoma cell line U251 was purchased from the American Type Culture Collection (ATCC, Manassas, VA, United States). Cells were cultured in Dulbecco’s Modified Eagle’s Medium (DMEM, 4.5 g/L L-1glucose, Lonza) supplemented with 10% fetal bovine serum (FBS, Gibco, Life Technologies, Budapest, Hungary), 4 mM l-glutamine, 100 U/ml penicillin, and 100 μg/ml streptomycin (Sigma-Aldrich, Budapest, Hungary) at 37°C containing 5% CO_2_.

### Irradiation Parameters

Irradiation was performed with an X-ray system, (RS320, Xstrahl Limited) at the Extreme Light Infrastructure Attosecond Light Pulse Source (ELI-ALPS) Research Institute. 250 keV energy X-ray beam was used to irradiate the cells seeded into 6- and 96-well plates at a dose rate of 3.65 Gy/min. The plates were placed into a special polymethyl methacrylate (PMMA) slab phantom [28], with the isocenter positioned at the geometrical centers of the plates. The delivered doses (0, 2, 5, and 8 Gy) were verified by performing dosimetry measurements in a regular manner using a calibrated ionization chamber (Farmer type ionization chamber–PTW TM30013), film dosimetry (Gafchromic EBT self-developing dosimetry film) and modified FBX type dosimeters prepared in our laboratory [28]. The combined standard uncertainty of the measurements was 2.2%. For each irradiated plate, control measurements were performed using Gafchromic EBT3 films.

### Basic Colorimetric Viability Assay

The viability of U251 cells was determined using a colorimetric MTT (3-(4,5-dimethylthiazol-2-yl)-2,5-diphenyltetrazolium bromide) assay. The cells with a density of 2,500, 5,000, and 10,000 cells/well, were seeded into 96-well tissue culture plates and cultured overnight prior to irradiation. 72 h after irradiation, 20 μl MTT solution (5 mg/ml) (Thermo Fisher Scientific United States) was added to each wells, and the plates were incubated at 37°C in an atmosphere containing 5% CO_2_ for 3 h. Following incubation, acid isopropanol (100 µl of 0.04 N HCl in isopropanol) was added to the wells and mixed thoroughly to dissolve the dark purple crystals. Subsequently, 20 μl 10% sodium-dodecyl-sulfate was added to the wells and after a few minutes, the absorbance of the plates at room temperature (RT) was measured using a plate reader (Ensight Multimode Plate Reader, PerkinElmer, United States) at a wavelength of 540 nm, with the values expressed in arbitrary units. All MTT cell viability experiments were performed at different cell densities in triplicate. The cell viability was calculated using the following equation:

$$\text{Cell viability }\left(\%\right)\;=\;\left({\text{Abs}}_{\text{sample}}/{\text{Abs}}_{\text{control}}\right)\;\times \;100,$$

where Abs_sample_ is the absorbance of the treated cells and Abs_control_ is the absorbance of the control cells (incubated with cell culture medium only).

### Colony Formation Assay

Colony formation assay was performed on the cells irradiated at different dose levels (0, 2, 5, and 8 Gy). The cells were grown in flasks with a surface area of 75 cm^2^, until they reached 75% confluence. For irradiation, the cells were subsequently seeded at 1,000 cells/well into 6-well plates, and were maintained and incubated for 8 days. From the naturally formed colonies with more than 50 cells, the medium was poured off and the cells were washed with phosphate-buffered saline (PBS) prior to fixation with 4% formaldehyde for 15 min. Colony forming units (CFUs) were stained with a solution containing 0.5% (w/v) crystal violet solution (Acros Organics, United States) for 20 min. After the staining procedure, the plates were rinsed three times with tap water and left to dry at RT. The number of colonies was counted under a microscope (Zeiss Axiovert 25, Zeiss, Germany), with the number of CFUs expressed as a percentage of the control samples, which were considered 100%. All colony forming assay experiments were performed in triplicate.

### Flow Cytometry

The cell cycle distribution and pγ-H2AX content were assayed simultaneously within one tube. The cell cycle and the Sub-G1 fraction were assayed as described previously with some modifications [29]. Briefly, the U251 cells (2 × 10^5^) were plated in 6-well dishes (Corning Life Sciences) and were irradiated with 2 Gy, 5 Gy or were left untreated. After 24 h or 72 h, the supernatants (S/N) were harvested into 5 ml conical tubes (Greiner). Cells were washed with 500 µl PBS, then PBS was pooled into the corresponding conical tubes. Cells were detached by 500 µl trypsin-EDTA (Lonza) at 37°C for 5 min. Trypsin was blocked with 20 µl FCS (Euroclone). Cells were pipetted into the corresponding conical tubes. Wells were washed with 500 µl PBS, and PBS was pipetted into the corresponding conical tubes. Cells were pelleted by centrifugation at 350 *g* for 6 min. The S/N was removed, cells were washed with 1 ml PBS, and centrifuged at 350 g for 6 min. S/N was removed, cells were suspended in –20°C cold 1.5% formaldehyde (Molar Chemicals) and incubated for 15 min at –20°C. Cells were centrifuged at 350 g for 6 min, S/N was removed. Cells were suspended in 4 ml −20°C cold 80% EtOH (Molar Chemicals) and incubated at –20°C for 1 h. Cells were centrifuged at 350 g for 6 min, S/N was removed. Cells were resuspended in 1 ml FT-PBS (PBS containing 1% FCS and 0.2% TritonX-100). Cells were centrifuged at 350 g for 6 min, S/N was removed. Cells were resuspended in 1 ml FT-PBS and incubated at RT for 5 min. Cells were centrifuged at 350 g for 6 min, S/N was removed. Cells were resuspended in 100 µl FT-PBS containing 1:1,000 dilution of anti-pγ-H2AX antibody (MA1-2022, Thermo Fisher Scientific) in dark conditions at RT for 45 min. Cells were washed 2 times with 1 ml FT-PBS, centrifuged at 350 g for 6 min, S/N was removed. The technical control for the “second antibody only” sample was left unstained with the primary antibody. Cells were resuspended in 50 µl FT-PBS containing 1:500 dilution of anti-mouse-Alexa 488 secondary antibody (A-11001, Thermo Fisher Scientific) in dark conditions at RT for 20 min. The antibodies were titrated in our laboratory to minimize background and increase sensitivity labeling U251 cells. Cell were washed with 1 ml FT-PBS, centrifuged at 350 g for 6 min, S/N was removed. Cells were stained with 300 µl DNA staining solution (DSS, PBS containing 0.1% Na-citrate, 10 μg/ml propidium iodide (PI), 0.1% TritonX-100, 10 μg/ml RNase A) and incubated in dark conditions at RT for 20 min. Cells were acquired on a Cytoflex S (Beckman Coulter) flow cytometer (FACS) in the following channels: excitation 561 nm, filter: 585/43 for PI; and excitation 488 nm, filter 525/40 for the Alexa 488. The FCS files were analyzed in CytExpert and Kaluza software (Beckman Coulter), data were visualized in GraphPad Prism 8.0. Experiments were performed in triplicates, and the arithmetic means, and standard deviations were calculated.

### Immunofluorescence Staining

For the analysis of DNA double strand breaks (DNA-DSB), the cells were seeded onto 22 × 22 mm coverslips at a thickness of 170 ± 5 µm suitable for confocal and superresolution microscopy. After 24 h of incubation, the cell layer was irradiated. The end of dose delivery was considered as 0 time point. After irradiation, the cells were washed twice with PBS, and then fixed with 4% paraformaldehyde (Sigma-Aldrich) at 30 min, 24 and 72 h postirradiation for 10 min. This step was followed by another PBS wash, after which the cells were permeabilized with 0.25% Triton X-100/PBS for 10 min. Thereafter, the cells were blocked with 1% bovine serum albumin/normal goat serum/phosphate buffered saline (BSA/NGS/PBS) (Sigma-Aldrich) and incubated at RT for 60 min. The cells were incubated with primary antibodies diluted in 0.5% NGS/PBS: primary mouse phospho-histone H2AX (Ser 140) monoclonal antibody (3F2, Thermo Fisher Scientific, United States) was used in 1:330 dilution and incubated for 60 min at RT. After the washing steps, the following secondary antibody was used: Alexa Fluor 647 conjugated anti-mouse IgG (Thermo Fisher Scientific, United States) in 1:500 dilution incubated at RT in dark conditions for 60 min. Nuclei were visualized with 4’,6-diamidino-2-phenylindole (DAPI, Sigma-Aldrich) staining.

### Confocal Microscopy

Confocal images were captured with a Nikon C2+ confocal scan head attached to a Nikon Eclipse Ti-E microscope. Confocal images were captured using a high NA, PLAN 60X objective (Nikon CFI Plan Apo Lambda 60X Oil with NA = 1.4), which enabled us to create high resolution images with a larger number of cell nuclei in the FOV throughout the experiments than during dSTORM measurements. The setup and data acquisition process were controlled by the Nikon NIS-Elements 5.02 software and the captured images were postprocessed in MATLAB. The Nikon Laser Unit was used to set the wavelengths and the power of the applied lasers operated at 405 nm (P_max_ = 60 mW; Nichia) and 647 nm (2RU-VFL-P-300-647-B1, P_max_ = 300 mW, MPB Communications Ltd).

All DSBs induced in the cell nuclei (N = 49–125) were displayed and counted in 3D.

### Analysis of Confocal Measurements

**FIGURE 1:**
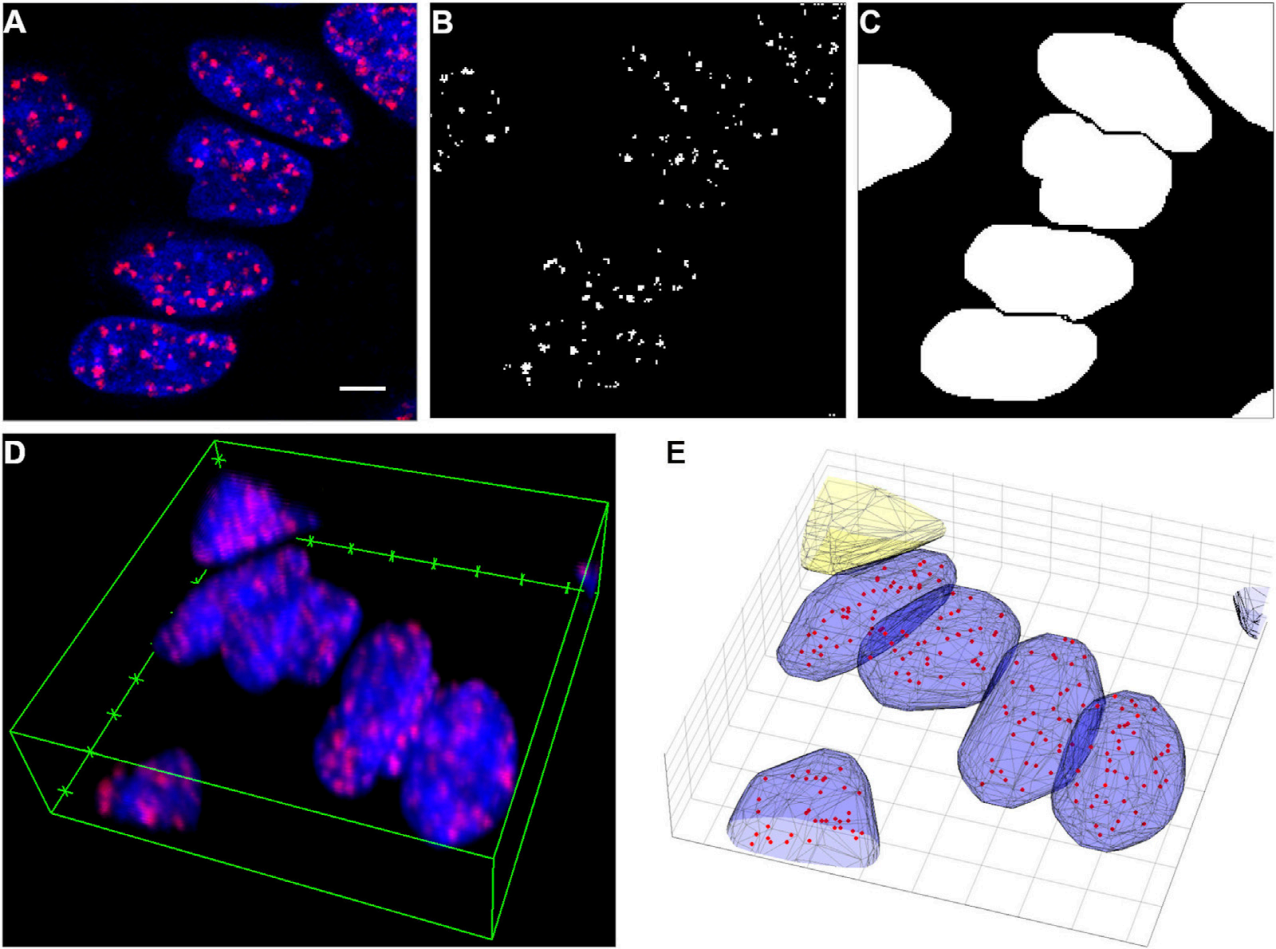
**(A)** A selected area from a confocal image slice (red: Alexa Flour 647-γH2AX, blue: DAPI-DNA). **(B)** Segmented γH2AX clusters in the area shown in Figure **(A)**. **(C)** Segmented nuclei in the same area. **(D)** The whole volumetric image of the area shown in Figure **(A)**. **(E)** Segmented nuclei (blue), a filtered out object (yellow–protruding) and γH2AX clusters (red dots). Scalebar: 5 µm.

A thresholding process based on the maximum entropy method [30] was applied on the confocal images (Figure 1A) to separate the γ-H2AX clusters from the background (Figure 1B). In this method, clusters are formed by the connected voxels. The centers and volumes of such clusters were determined and used as a merit function of the quantitative evaluation. Small clusters (<5 voxels = 0.06615 µm^3^) were considered as noise and were excluded from further statistics.

The signal of DAPI was used to contextualize these clusters. The nuclei were segmented using a watershed algorithm (Figure 1C) [31]. This algorithm can determine the separating lines (ridge lines) between connected objects, but it requires preprocessing of the images.

After contrast adjustment, the images were filtered with a Gaussian smoothing kernel with a standard deviation of 2 pixels. The image slice containing the higher signal (sum of all pixel values) was used to calculate a global threshold (the mean of the locally adaptive threshold values gained with the Bradley’s method [32]) for binarization.

A dilation algorithm was applied with a structuring element of a disk (radius = 2 pixels) in order to make the nuclei more visible. The holes that possibly remained inside the nuclei were filled with a flood-fill operation [33]. Morphological opening was also performed in order to eliminate small blobs from the images. This contains an erosion and a dilation step with a structuring element of a disk (radius = 10 pixels). A distance transform based on a fast algorithm [34] was performed, which produced a local intensity minimum in place of each cell nucleus. The watershed transform has a tendency to oversegment, therefore the tiny local minima from this distance transform were eliminated. This was performed by computing the extended-minima transform, which produced small spots that were roughly in the middle of the nuclei to be segmented, and by modifying the distance transform with the MATLAB function called imimposemin. Then the watershed algorithm found the ridge lines on these processed images, which could in turn be used to segment the nuclei. It proved efficient to segment the nuclei in each image slice separately and then repeat the protocol in the compiled 3D volumetric binary images (Figure 1D). The identified objects were filtered based on their properties (volume, surface/volume ratio) or their position (for example protruding from the image volume) (Figure 1E).

Finally, the γ-H2AX clusters were associated with these identified nuclei in order to determine the cluster number per cell and cluster density distribution.

### dSTORM Microscopy

Superresolution dSTORM measurements were performed on a custom-made inverted microscope based on a Nikon Eclipse Ti-E frame with an oil immersion objective (CFI Apo TIRF 100XC Oil, NA = 1.49). EPI-fluorescence illumination was applied at an excitation wavelength of 647 nm. The laser intensity was set to 2–4 kW/cm^2^ on the sample plane and controlled via an acousto-optic tunable filter. A filter set from Semrock (Di03-R405/488/561/635-t1-25 × 36 BrightLine^®^ quad-edge superresolution/TIRF dichroic beamsplitter and FF01-446/523/600/677-25 BrightLine^®^ quad-band bandpass filter, and an additional AHF 690/70 H emission filter) was inserted into the microscope to spectrally separate the excitation and emission lights. The images of individual fluorescent dye molecules were captured by an Andor iXon3 897 BV EMCCD camera (512 × 512 pixels with 16 μm pixel size) with the following acquisition parameters: exposure time = 30 ms; EM gain = 200; temperature = −75°C. Typically 20,000 to 50,000 frames were captured from a single ROI. During the measurement, the Nikon Perfect Focus System kept the sample in focus. High-resolution images were reconstructed with the rainSTORM localization software [35–38]. Spatial drift introduced by either the mechanical movement or thermal effects was analyzed and reduced by means of an autocorrelation-based blind drift correction algorithm.

### dSTORM Buffer

The dSTORM experiments were conducted in a GLOX switching buffer [39] and the sample was mounted onto a microscope slide. The imaging buffer is an aqueous solution diluted in PBS containing an enzymatic oxygen scavenging system GluOx (2000 U ml^−1^ glucose-oxidase (Sigma Aldrich, catalog number: G2133-50KU), 40 000 U ml^−1^ catalase (Sigma Aldrich, catalog number: C100), 25 mM potassium chloride (Sigma Aldrich, catalog number: 204439), 22 mM tris (hydroxymethyl) aminomethane (Sigma-Aldrich, catalog number: T5941), 4 mM tris (2-carboxyethyl) phosphine (TCEP) (Sigma-Aldrich, catalog number: C4706) with 4% (w/v) glucose (Sigma Aldrich, catalog number: 49139) and 100 mM β-mercaptoethylamine (MEA) (Sigma-Aldrich, catalog number: M6500). The final pH was set to 7.4.

### Analysis of dSTORM Measurements

The γ-H2AX clusters were identified *via* a clustering algorithm based on DBSCAN [40]. The convex hull of the clusters was considered as the cluster area, and the position of the clusters were associated with the geometric center of the localization coordinates, which was an important property for the calculation of the cluster density distribution inside the nuclei. The nuclei areas were determined using the conventional fluorescent images taken during the dSTORM measurements and the segmentation approach described in *Analysis of Confocal Measurements*. The number of epitopes per cluster was estimated by fitting the localization number histogram of the clusters belonging to individual epitopes with a theoretical model [25, 41]. This merit function was calculated for different regions of the image because the photophysics of the used fluorophores depends on the intensity of illumination, which was not homogeneous throughout the FOV.

## Results

### Cell Viability and Colony Forming Assays

In order to define the standard dose response curve and the data which can be derived from them, furthermore to apply the optimal parameters for the experiments (cell numbers, radiation doses and detection time points), MTT and colony forming assays were performed. The standard MTT assay manifested the expected dose dependent decrease in cell viability assessed at 72 h postirradiation (Supplementary Figure S1A). The clonogenic formation assay confirmed the known radiation sensitivity of the U251 cell line (Supplementary Figure S1B).

### Combined Cell Cycle and pγ-H2AX Analysis With Flow Cytometry

**FIGURE 2:**
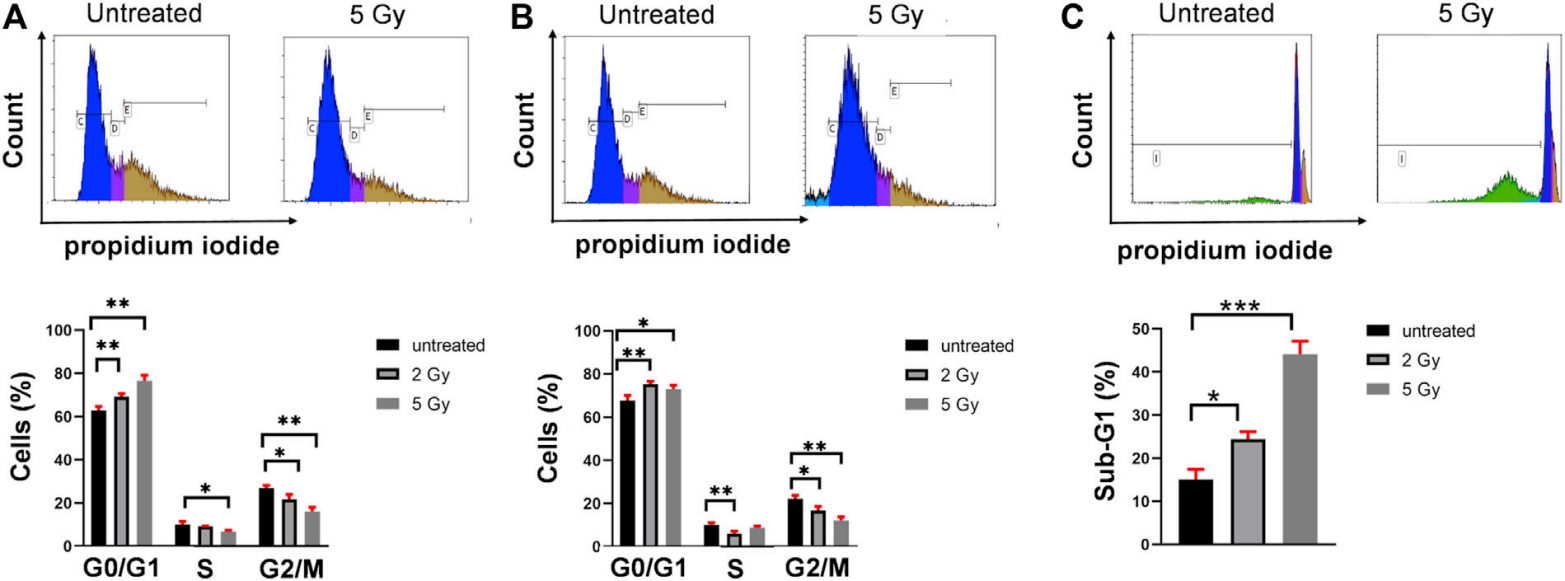
Irradiation induces cell cycle arrest in G0/G1 phase and apoptosis of U251 cells. **(A)** Representative dot plots of cell cycle analysis after 24 h (upper part). Irradiation caused significant G0/G1 cell cycle arrest, decrease both in S and G/2M phase (lower part) after 24 h. **(B)** Representative dot plots of cell cycle analysis after 72 h (upper part). Irradiation caused significant G0/G1 cell cycle arrest, decrease both in S and G/2M phase (lower part) after 72 h. **(C)** Representative dot plots of Sub-G1 analysis after 72 h (upper part). The internucleosomal DNA fragmentation increased upon 2 and 5 Gy irradiation after 72 h *p <0.05, **p <0.01, ***p <0.001.

Using the propidium iodide DNA intercalator and flow cytometry technology we investigated the effect of 2 and 5 Gy irradiation to the cell cycle phases of U251 cells. The irradiation of U251 cells caused significant cell cycle arrest in G0/G1 phase at the expense of the decrease of S and G2/M cell cycle phases measured after 24 h or 72 h postirradiation (Figures 2A,B). The final apoptotic event, the internucleosomal DNA fragmentation was assayed after 72 h. The hypodiploid Sub-G1 population increased at 2 Gy (24%) and 5 Gy (44%) irradiation after 72 h (Figure 2.C).

Combined flow cytometric single cell analysis was applied to measure both the cell cycle and the percentage of pγ-H2AX+ U251 cells in the same sample of asynchronous cell culture. Using the propidium iodide DNA intercalator and flow cytometry technology we investigated the effect of 2 and 5 Gy irradiation to the cell cycle phases of U251 cells. The irradiation of U251 cells caused significant cell cycle arrest in the G0/G1 phase at the expense of the decrease of S and G2/M cell cycle phases measured after 24 h (Figure 2A) or 72 h postirradiation (Figure 2B). The final apoptotic event, the internucleosomal DNA fragmentation was assayed after 72 h. The hypodiploid Sub-G1 population increased at 2 Gy (24%) and 5 Gy (44%) irradiation after 72 h (Figure 2C).

**FIGURE 3:**
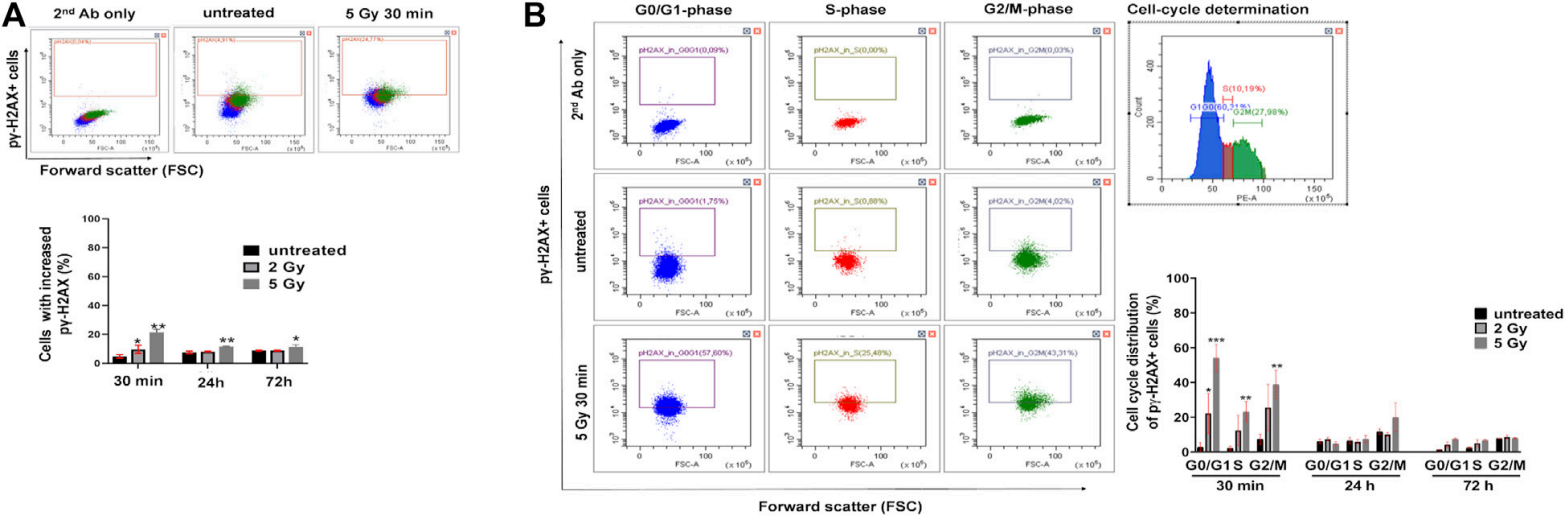
Irradiation induces the increase of pγ-H2AX+ cells in U251 cells. **(A)** Representative dot plots of pγ-H2AX+ immunofluorescent staining detected by flow cytometry (upper part) and the percentage of U251 cells with the increase of pγ-H2AX signal (lower part) following irradiation. **(B)** Representative dot plots of the distribution of pγ-H2AX+ U251 cells within the G0/G1, S, and G2/M cell cycle phases, separately (left panel). Representative cell cycle distribution of untreated cells (right panel). Irradiation caused significant increase of pγ-H2AX+ U251 cells both within G0/G1, S, and G2M phases after 30 min (right lower graph). *p <0.05, **p <0.01, ***p <0.001.

Flow cytometry single cell analysis was applied to measure the percentage of U251 cells with increased pγ-H2AX in asynchronous culture. Thirty minutes after irradiation with 2 Gy or 5 Gy, the number of pγ-H2AX positive cells increased by 9.7 ± 2.7% and 21.4 ± 2%, respectively (Figure 3A). A smaller increase was detected in the percentage of pγ-H2AX positive cells with 5 Gy irradiation after 24 h (11.6 ± 0.29%) or after 72 h (11.42 ± 1.42%), respectively (Figure 3A). In order to reveal the cell cycle distribution of pγ-H2AX positive cells, we performed both the cell cycle analysis and pγ-H2AX measurement from the same samples. The pγ-H2AX positive cells were plotted from the G0/G1, S, and G/M cell cycle phases (Figure 3B). Thirty minutes after irradiation with 2 Gy, the pγ-H2AX positive cells showed the following distribution: 22% G0/G1, 12% S, or 25% G2/M cell cycle phase. The same parameters after irradiation with 5 Gy equaled 54% G0/G1, 23% S, or 38% G2/M cell cycle phase (Figure 3B). After 24 h or 72 h, no significant change was observed in the cell cycle phase distribution of pγ-H2AX positive cells (Figure 3B).

### γ-H2AX Analysis With 3D Confocal Microscopy

**FIGURE 4:**
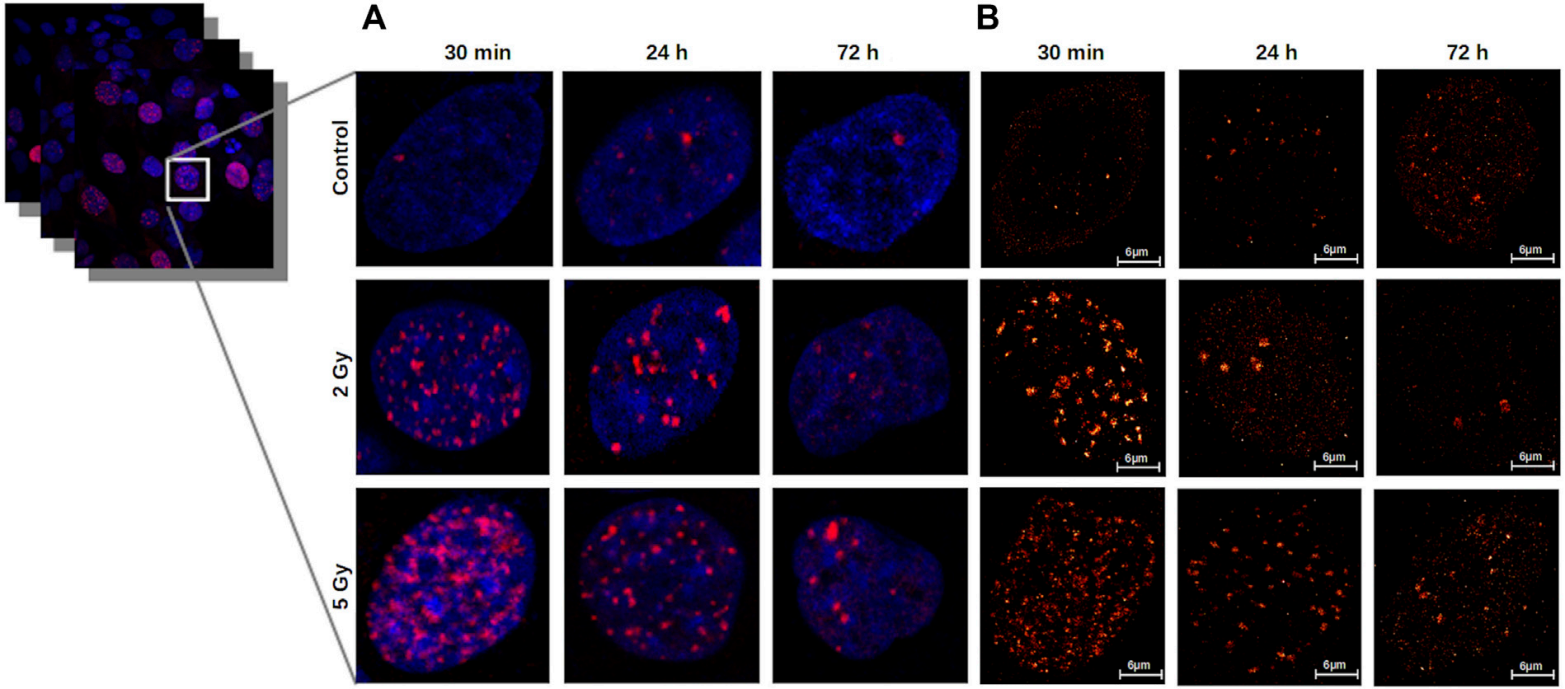
**(A)** Confocal images represent repair foci in U251 cell nuclei. 30 min after irradiation, the median number of γ-H2AX foci per cell increased significantly to a 15 times higher level than in the unirradiated cells. At 24 h, the foci number decreased in the 2 Gy group, but remained four times higher than the control, and in the 5 Gy group, a higher portion of DSBs remained unrepaired. **(B)** Detection and characterization of γ-H2AX clusters by dSTORM in U251 cell nuclei. The dSTORM images show the substructures of the repair foci. After irradiation with 2 Gy or 5 Gy, the cluster number and area reach their maxima at 30 min, and decrease at24 h and 72 h in a dose dependent manner. Scalebar: 6 µm.

The γ-H2AX clusters after different irradiation doses at different time-points were first measured by confocal microscopy as demonstrated in Figure 4A.

The median number of γ-H2AX foci in the control cell nuclei was 4 (25th and 75th percentiles are 0 and 15) (Supplementary Figure S2). The median number of γ-H2AX foci per cell increased 30 min after 2 Gy to 50 (p25: 35, p75: 64) and after 5 Gy to 43 (p25: 26.5, p75: 56.5), respectively. At 24 h, in the 2 Gy irradiated cells the number of γ-H2AX foci decreased to 12 (p25: 2, p75: 23.5), which is four times higher than 3 (p25: 1, p75:15.25) in the control. At 24 h after 5 Gy, a higher portion of DSBs remained unrepaired (30 foci/cell).

At 72 h postirradiation, the median of the number of γ-H2AX foci reached the control level in the nuclei of 2 Gy irradiated cells and was slightly higher than the control at 5 Gy dose level.

**FIGURE 5:**
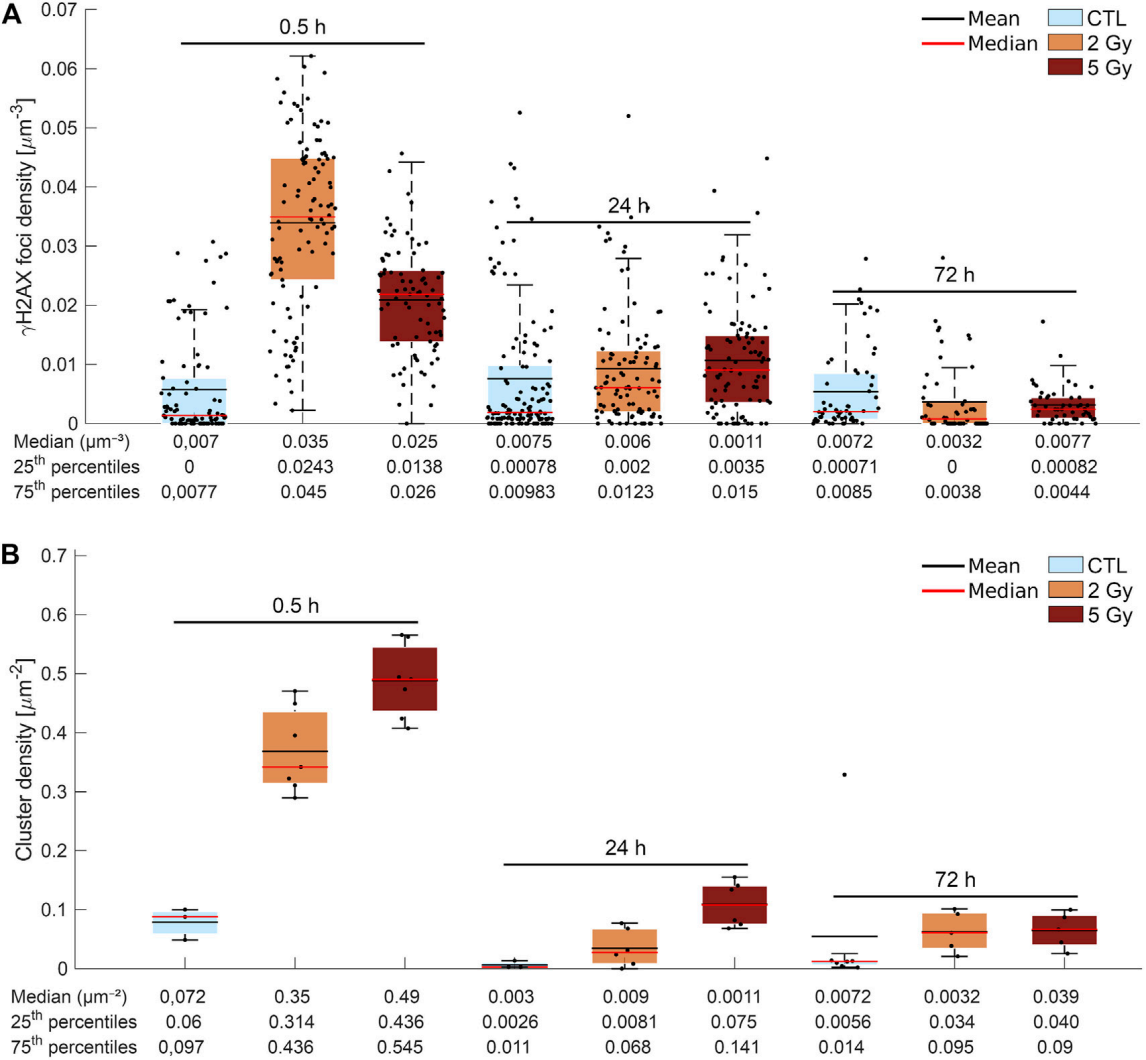
**(A)** Graphic representation of the confocal image analysis. Mean (black line) and median (red line) density (foci per µm^2^) of γ-H2AX foci/cell irradiated at different dose levels (control (blue), 2 Gy (yellow), 5 Gy (red) and fixed at the defined time points after treatment (0.5, 24, and 72 h). The median density of γ-H2AX foci was five times higher than the control 30 min after 2 Gy and lower, and only four times higher after 5 Gy. At 24 h, the density of γ-H2AX foci was still elevated, and at 72 h it decreased to the control value or below at both dose levels. **(B)** Evaluation of dSTORM images. The figure shows the cluster density (the number of DNA DSB repair foci per µm^2^) in the control and in the irradiated (2 Gy,5 Gy) cells 0.5, 24, and 72 h after treatment (mean: black line, median:red line). The cluster density (cluster number/cell nucleus area) increased considerably 0.5 h after x-ray irradiation, in a clear correlation with the dose level. At 24 h, the measured density remained three times higher after 2 Gy and six times higher after 5 Gy than in the control. A small amount of residual foci could be detected in both dose groups at 72 h.

The median density of γ-H2AX foci (number of γ-H2AX foci per cell volume) was 5 times higher than the control 30 min after 2 Gy and 4 times higher after 5 Gy, respectively (Figure 5A). At 24 h, the density of γ-H2AX foci did not drop to the control level: it equaled 0.006 µm^−3^ after 2 Gy and 0.011 µm^−3^ after 5 Gy, respectively. At 72 h postirradiation, the density of γ-H2AX foci decreased to the control value or below at both dose levels.

The confocal analysis showed fewer foci at higher doses (Figure 5A; Supplementary Figure S2) which seemed to contradict the images (Figure 4A). The anomalous result may be the outcome of the limited spatial resolution of the confocal technique. For this reason, high cluster density could cause merged foci, which reduces the detected foci number. This also means a growth in the size of detected foci, therefore, we analyzed the distribution of foci size.

**FIGURE 6:**
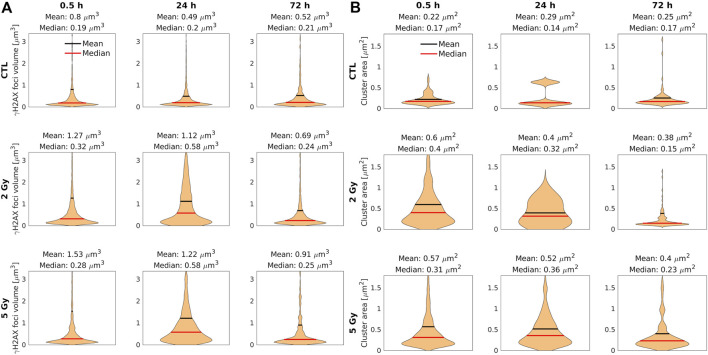
Violin plots represent the foci volume and cluster area of cell nuclei with confocal **(A)** and dSTORM **(B)** analysis. The median (red line) and the mean (black line) values of the γ-H2AX foci volume (A) and cluster area (B) in the control and the treated samples at 0.5, 24, and 72 h after irradiation. The foci volume **(A)** and cluster area **(B)** are smaller at 30 min after irradiation with 5 Gy, and larger after irradiation with 2 Gy. At 24 h after irradiation, the foci volume **(A)** and cluster area **(B)** increased compared to the values measured at 0.5 h min after irradiation.

In Figure 6A, the mean volume at 0.5 h postirradiation was 1.27 µm^3^ after 2 Gy, and 1.53 µm^3^ after 5 Gy, respectively. With time, the mean value of the γ-H2AX foci volumes decreased, however the samples treated with a higher dose (5 Gy) contained large γ-H2AX foci even after 72 h.

The statistical analysis of the cluster volumes supported our hypothesis about the merged foci. Few but large clusters can increase the mean value at 0.5 h postirradiation after 5 Gy, without changing the median value significantly. Because of this limit of the confocal technique, the examination of the foci requires more precise methods.

### γ-H2AX Analysis With dSTORM Microscopy

In addition to confocal images, nanoscale resolution became available with the advent of superresolution microscope technology [42, 43]. In contrast with the diffraction-limited confocal microscope images, in which γ-H2AX foci appear as homogeneous fluorescent spots (Figure 4A), the dSTORM images reveal the substructures of the repair foci (Figure 4B), and the localization data can be directly used for cluster analysis. Indeed, the nanometer resolution of the dSTORM gives us the capability to perform cluster analysis at foci level, which leads to the isolation of clusters inside the γ-H2AX foci. Although dSTORM is more precise than the confocal technique, but it is difficult to acquire images from a large number of cells. Therefore, it is a promising technique for correlative measurements. The analyzed cell numbers with dSTORM measurements were between 3 and 7 (mean: 5.44).

The cluster size represents the number of fluorophore localizations, and is determined by the staining protocol and the imaging resolution. Considering the antibody size and the localization precision, the apparent size of a labeled structure is about 40–50 nm. The cluster analysis of images opened the way towards the quantitative evaluation of individual foci. After the response function is determined, several parameters (epitope number, cluster area etc.) can be analyzed statistically.

Assessment of the cluster density of γ-H2AX (number of foci per unit area) from the nucleus center to the nucleus envelope in five areas yielded equal distributions irrespective of dose and time point (Supplementary Figure S3). This is supported by nearest‐neighbor‐distances (NNDs) within the cell nuclei with a median value of about 170 nm (164–200).

In Figure 5B, cluster density (cluster number/cell nucleus area) increased drastically 0.5 h after x-ray irradiation, exhibiting a clear correlation with the dose: 0.35 μm^2^ at the 2 Gy dose level, and 0.49 µm^2^ at the 5 Gy dose level. In terms of cluster density, we observed different dynamics in the return to the normal (endogenous) level: at 24 h, the measured density was three times higher after 2 Gy and six times higher after 5 Gy than in the control (0.09 μm^2^). Some residual foci could be detected even 72 h after irradiation, irrespective of the applied dose. The distribution of the γ-H2AX foci showed a random distribution inside the nuclei and no difference was found between the endogenous (control) and the irradiated (treated) samples in this regard (Supplementary Figure S3).

From Figure 5B to Figure 6B we can conclude that 30 min after irradiation with 5 Gy, the area of clusters is smaller while the number of clusters is higher compared to irradiation with 2 Gy. The density and sizes of the cluster area suggest that the coverage of nucleus by clusters is almost the same, therefore we quantified the sum of clusters area per cell nucleus area (data not shown). This cluster coverage was higher 30 min after 5 Gy irradiation (median (Md): 0.28254), but showed no marked difference compared to 2 Gy (Md: 0.2127). Therefore, the amount of phosphorylated γ-H2AX histones is almost similar between the 2 and 5 Gy groups, 30 min postirradiation, however its distribution is different.

**FIGURE 7:**
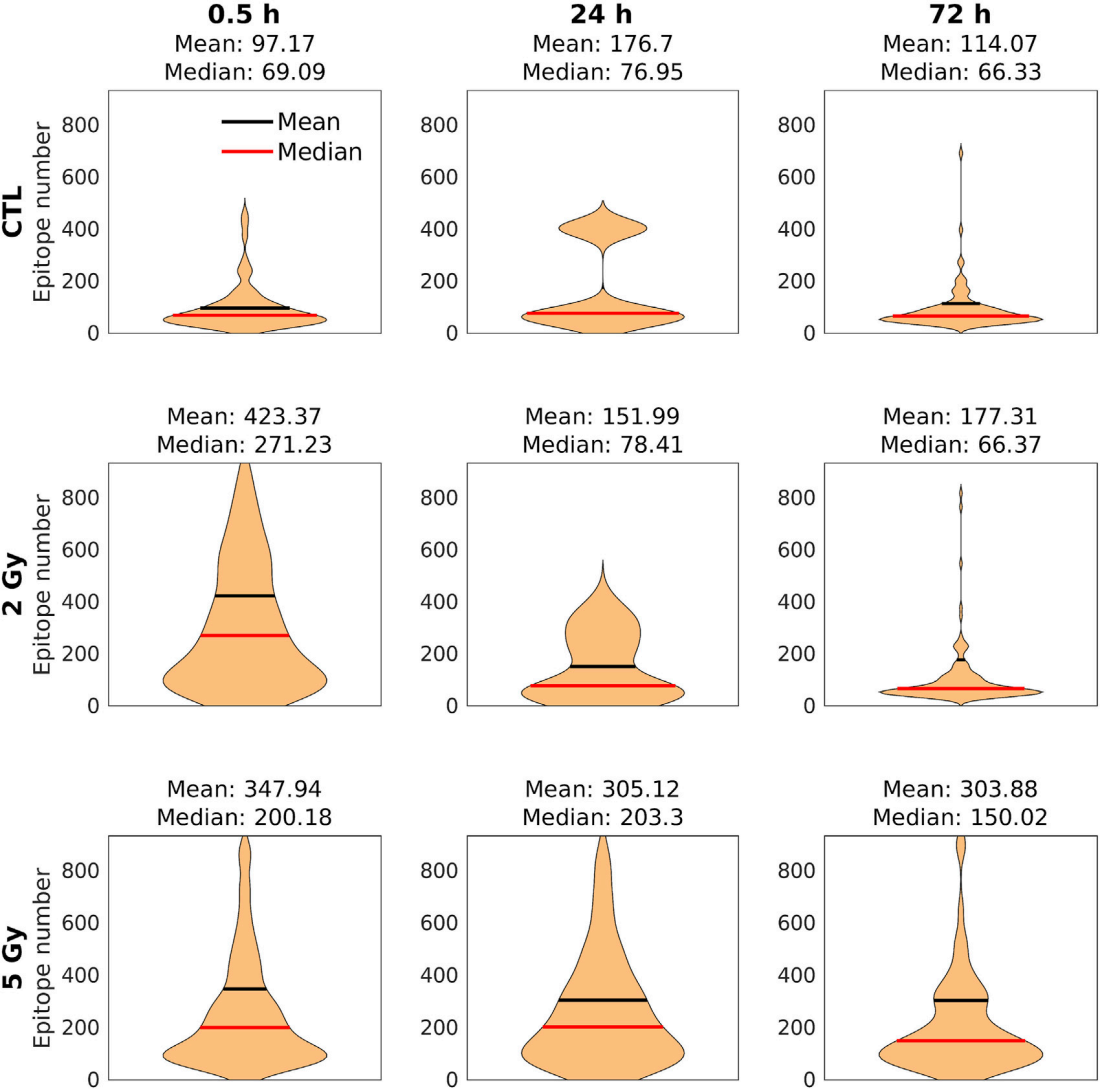
The distribution of the epitope number/clusters. Black lines: mean values; red lines: median values. There was a significant increase in the mean of epitopes/clusters over the control both after 2 and 5 Gy. At 24 h, it decreased to the control level and stayed there at 72 h in the 2 Gy group. However, in the 5 Gy irradiated group we could observe a very slow decrease by 72 h, when a considerable number of epitopes could still be detected.

Localization data can be applied for a more detailed evaluation of clusters to determine the area of the clusters (Figure 6B) and the epitope number/cluster (Figure 7). Both parameters follow similar trends and their mean (black) and median (red) values can be used for the quantitative evaluation of the repair mechanisms. The median area of the clusters at 0.5 h postirradiation was 0.40 µm^2^ after 2 Gy, and 0.31 µm^2^ after 5 Gy (Figure 6B). At the 24 h observation point, the median area of the foci in both irradiated samples (0.32 µm^2^ after 2 Gy and 0.36 µm^2^ after 5 Gy) was double the control value. At 72 h postirradiation, the cluster area did not differ from the control (0.15 versus 0.17 µm^2^) after 2 Gy, but it grew to 0.23 µm^2^ after 5 Gy.

In contrast with diffraction‐limited confocal microscope images, in which γ-H2AX foci appear as homogeneous fluorescent spots (Figure 4A), on the dSTORM images the foci clearly consist of spatially separated subunits with a diameter of 40–50 nm, corresponding to chromatin structures (Figure 4B). There were considerable differences in the average number of epitopes/clusters compared to the control (Md: 69.09) 30 min after 2 Gy (Md: 271.23) and 5 Gy irradiation (Md: 200.18) (Figure 7). After 24 h, the mean epitope number decreased to the control level in the 2 Gy irradiated group, while a similar elevated value was observed in the 5 Gy irradiated group. At 72 h postirradiation, the residual clusters contained more epitopes than the foci in the control group, which was more pronounced in the 5 Gy group.

## Discussion

The studied human glioblastoma U251 cells showed radiosensitivity, namely hampered viability, G0/G1 cell cycle arrest and apoptotic DNA degradation upon irradiation. Cells were not synchronized before irradiation to model the clinically relevant cellular division at the time of irradiation. In eukaryotic cells, DNA is wrapped around the core histone molecules forming the nucleosome complexes, which are equally spaced subunits of the chromatin. Histone cores contain families of histone proteins H2A, H2B, H3, and H4 [44]. Among them, H2AX molecules, randomly incorporated in histones throughout the DNA, play a key role in sensing and initiating the DDRs. In order to investigate the individual γ-H2AX‐containing nucleosomes at DSB sites, the resolution of microscopic detection should be at the nanoscale [13, 45, 46]. In our study, we used 3D confocal microscopy and dSTORM to follow the temporal response up to 72 h and the effectiveness of DNA repair processes with high resolution after exposing glioblastoma cells to therapeutic doses of X-ray. The endogenous (non-treated) U251 cells may contain γ-H2AX foci, which can be explained by spontaneous replication errors occurring in rapidly proliferating cells, e.g., in several cancer cell lines [44, 47].

It has recently become evident that H2AX phosphorylation is important not only in signaling the DNA damage, but also in the chromatin remodeling process. Chromatin relaxation and remodeling from the compact chromatin network are important in DNA repair: they pave the way for repair proteins to access the damaged regions of the DNA. It has already been observed, that when confocal microscopy is used with stained cells, most of the γ-H2AX appear in the region above the mean DNA level in the irradiated cells [45, 46]. We revealed no special arrangement of the DSBs inside the nucleus: the foci, the individual γ-H2AX histones inside it and the sub 100 nm fibers were randomly spread throughout the nucleus [45]. Our dSTORM analysis could confirm the homogeneous distribution of γ-H2AX labeled chromatin in the nucleus. Sisario et al. conducted an investigation on DNA‐repair protein kinase including γ-H2AX foci analysis by superresolution microscopy, and revealed that the dSTORM‐based γ-H2AX foci counting and distance measurements between the foci provided quantitative information on the total amount of chromatin molecules involved in DSB repair, as well as on the number and longitudinal distribution of γ-H2AX‐containing nucleosomes in a chromatin fiber. We could also identify beadwork like nucleosome structures by dSTORM analysis [48].

It is well known that the level of γ-H2AX foci is linked to the delivered dose, depending on the cell line, cell proliferation, irradiation parameters such as dose rate and radiation quality [49]. H2AX phosphorylation reaches its peak value at around 30 minutes after radiation exposure. In our examination flow cytometric measurement confirmed the highest pγ-H2AX signal after 30 min postirradiation, and further analysis revealed the cell cycle phase distribution of these pγ-H2AX positive cells. The increase of pγ-H2AX positive U251 cells was shown both in the G0/G1, S, and G2/M cell cycle phases after 30 min irradiation with no cell cycle-dependency. With quantitative microscopy, we measured the maximum number and density of γ-H2AX foci 30 min post-irradiation at both dose levels, and the values showed good agreement with those published in literature [16]. However, advanced 3D confocal microscopy did not exhibit any correlation between the γ-H2AX foci number/density and the doses in the applied dose range. This can be attributed to the limitation of the confocal microscopy techniques if the γ-H2AX foci are too large and too high in number. Indeed, at high densities, as expected at high doses, the 20–60 nm foci overlap [46] and the cluster analyzer code merges the smaller structures into larger units. The evaluation of the volume of the detected clusters confirmed this assumption. Moreover, 24 hours later, the FACs analysis also showed the presence of pγ-H2AX, but only at 5 Gy, which indicates a significant dose dependent difference. This confirmed that at this time point the DDR was successfully finalized in the cells irradiated with 2 Gy, but relevant residual pγ-H2AX foci could be counted, which represented the unrepaired DSBs. At 72 h postirradiation, the pγ-H2AX level was still significantly higher in the highest dose group, suggesting a long lasting ongoing repair process in case of irradiation with 5 Gy. In contrast to diffraction limited detection by confocal microscopy, the dSTORM analysis confirmed the dose dependence in the amount of DSBs. Much smaller units, so called clusters, in the nanometer regime (hundreds of nm^2^ area) could be analyzed. At the higher dose level (5 Gy), clearly higher cluster density was detected and more clusters could be counted 30 min after irradiation, than at the lower dose level. Along these maximum γ-H2AX values, the area of the clusters and epitope number/clusters proved to be lower than from the 2 Gy exposure. A similar trend was observed on the basis of cluster coverage (sum of the cluster areas per cell nuclei area) and it also correlated with the density and epitope number per cluster. Thirty minutes after 5 Gy irradiation, cluster coverage was slightly higher but there was no marked difference after 2 Gy. Based on this, cluster coverage suggests that the amount of phosphorylated γ-H2AX histones is similar at 30 min postirradiation in the 2 and 5 Gy groups, but their distribution changes. This can be explained with the definite number of molecules participating in the repair processes. However, 24 h later, after the vast majority of the DSBs was successfully repaired, at the more serious damage sites active DDR was reinduced as represented by the residual γ-H2AX foci, which are larger in cells irradiated at the higher dose level. We observed the same trend regarding the epitope number: more epitopes were detected from the higher dose at both later time points, i.e., 24 and 72 h postirradiation. The distribution of NNDs proved to be independent of dose and postirradiation time [50, 51]. The introduction of 3D confocal microscopy in the analysis of DSBs represents a relevant step for a more realistic determination of the size, number and spatial arrangement of γ-H2AX foci over the commonly used epifluoroscope microscopy [52]. In contrast with diffraction limited confocal microscopy, dSTORM measurements ensured much higher accuracy for the quantitative analysis of DNA DSBs, and provided a more detailed insight at individual molecular level into the spatiotemporal processes of γ-H2AX formation and rearrangements, enabling comprehensive analysis of the short-term and slow repair processes of radiation induced DNA damage [53].

## Data Availability

The raw data supporting the conclusion of this article will be made available by the authors, without undue reservation.

## Supplementary Material

Supplementary Figure 1
**(A)** Viability of the U251 cells at 72 h following 0-2-5-8 Gy X-ray dose delivery. The data are expressed as the mean ± standard deviation. **p*<0.05, ***p*<0.01, ****p*<0.001 using the paired Student’s t-test; treated samples vs. 0 Gy (control) samples. **(B)** Clonogenic survival of U251 cells after 2 Gy, 5 Gy, and 8 Gy irradiation. The data are expressed as the mean ± standard deviation, and all experiments were performed in triplicate.

Supplementary Figure 2 Graphic representation of the mean (black line) and median (red line) of γ-H2AX foci numbers in cell nuclei irradiated at different dose levels (control–blue; 2 Gy–yellow; 5 Gy–red) and fixed at diverse time points after treatment (0.5, 24, and 72 h).

Supplementary Figure 3 The graphs show that the cluster density of γ-H2AX does not differ topologically, i.e., it shows equal distribution from the nucleus center to the nucleus envelope in theU251 cells irrradiated with 2 and 5 Gy, independently from the dose level. The observations were made at 0.5 h (orange), 24 h (blue) and 72 h (green) post-treatment.
