# Supplementary figures and images for: Analysis of Ionizing Radiation Induced DNA Damage by Superresolution dSTORM Microscopy
Source: Pathol Oncol Res. 2021 Nov 8;27:1609971. doi: 10.3389/pore.2021.1609971 (PMC8966514; doi:10.3389/pore.2021.1609971)

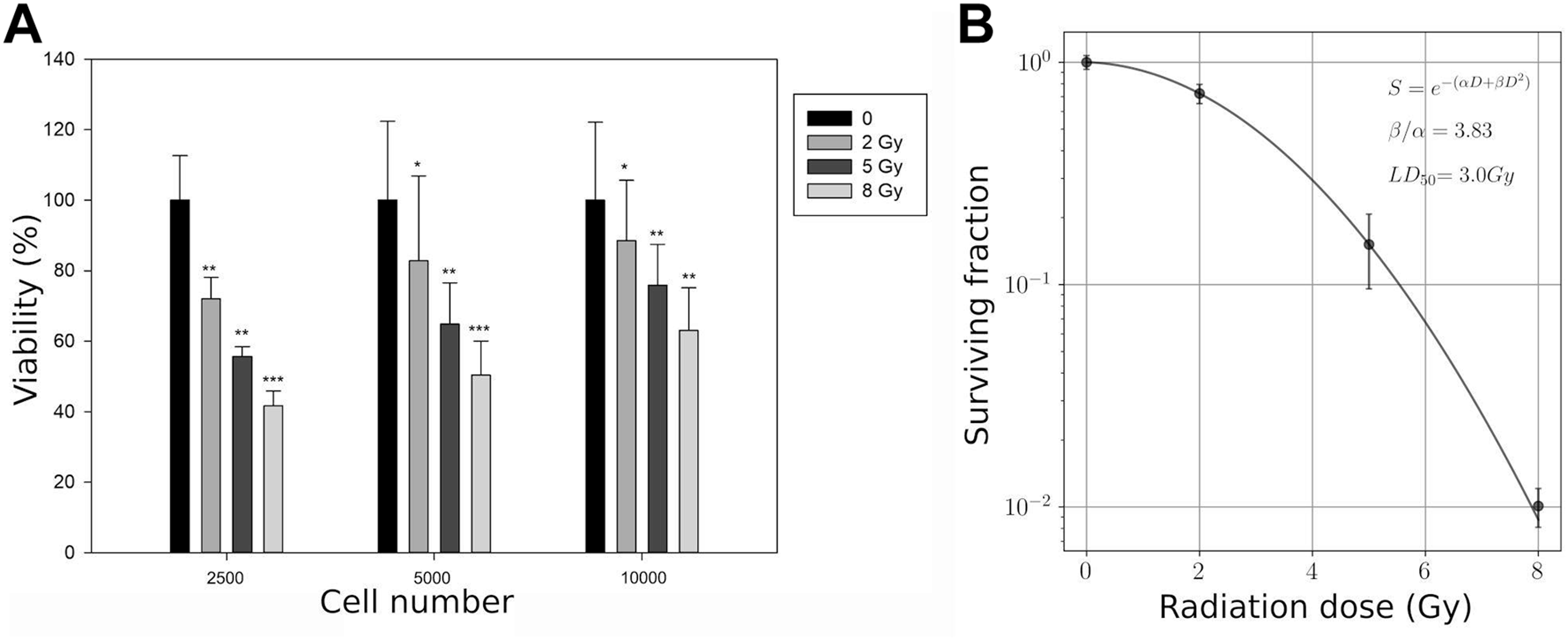

Supplement: Supplementary file 1 [file Image1.tif]

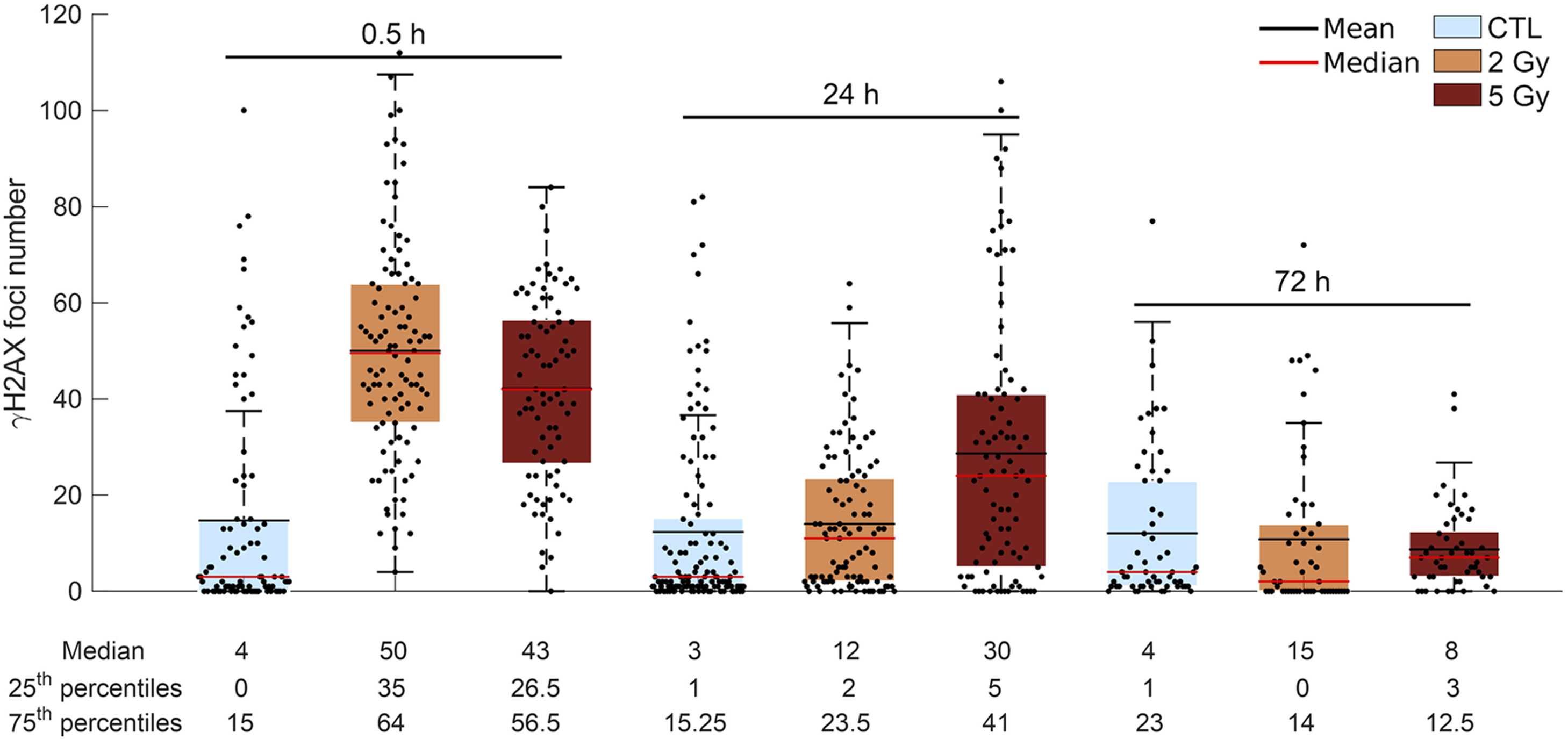

Supplement: Supplementary file 2 [file Image2.tif]

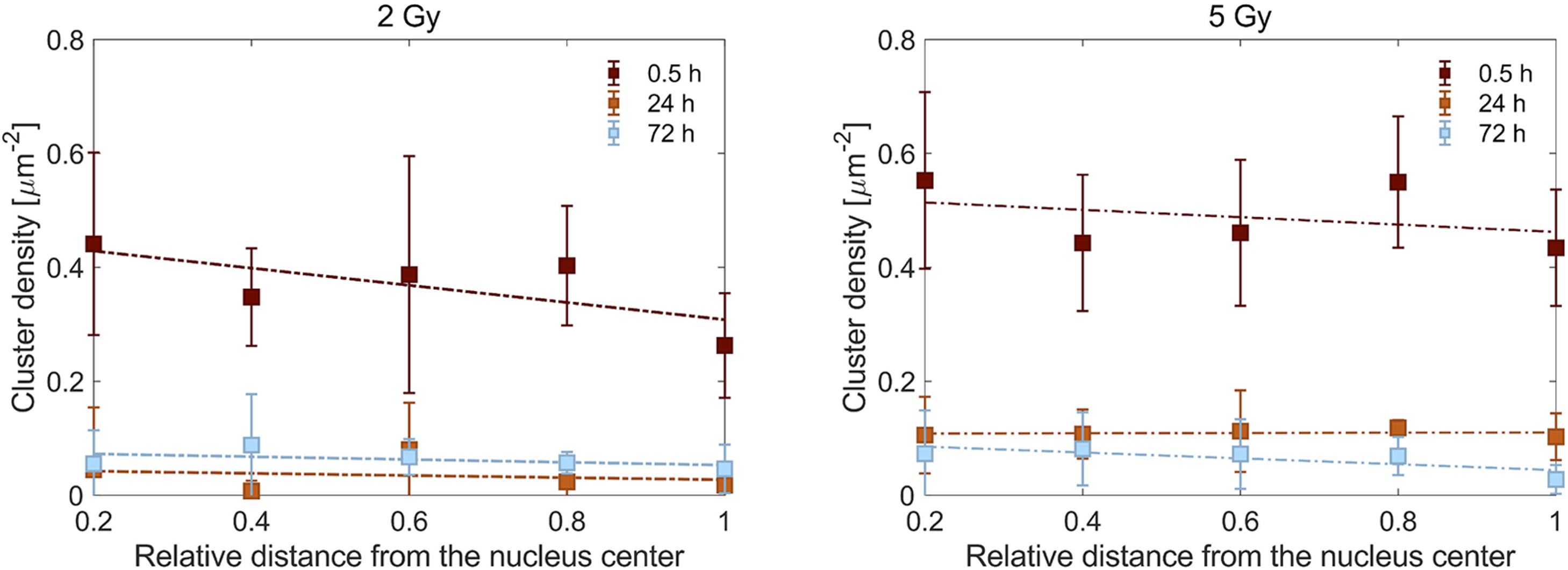

Supplement: Supplementary file 3 [file Image3.tif]
